# Treatment of multiple-level tracheobronchial stenosis secondary to endobronchial tuberculosis using bronchoscopic balloon dilatation with topical mitomycin-C

**DOI:** 10.1186/s12890-016-0209-1

**Published:** 2016-04-14

**Authors:** Mohamed Faisal, Hafaruzi Harun, Tidi M. Hassan, Andrea Y. L. Ban, Sanjay H. Chotirmall, Jamalul Azizi Abdul Rahaman

**Affiliations:** Department of Medicine, Respiratory Unit, Universiti Kebangsaan Malaysia Medical Centre, Jalan Yaacob Latiff, Bandar Tun Razak, 56000 Cheras Kuala Lumpur, Malaysia; Respiratory Unit, Serdang Hospital, Jalan Puchong, 43000 Kajang, Selangor Darul Ehsan Malaysia; Lee Kong Chian School of Medicine, Nanyang Technological University, Singapore, Singapore

**Keywords:** Case report, Endobronchial tuberculosis, Bronchoscopic intervention, Mitomycin-C

## Abstract

**Background:**

Tracheobronchial stenosis is a known complication of endobronchial tuberculosis. Despite antituberculous and steroid therapy, the development of bronchial stenosis is usually irreversible and requires airway patency to be restored by either bronchoscopic or surgical interventions. We report the use of balloon dilatation and topical mitomycin-C to successful restore airway patency.

**Case presentation:**

We present a 24-year old lady with previous pulmonary tuberculosis and laryngeal tuberculosis in 2007 and 2013 respectively who presented with worsening dyspnoea and stridor. She had total left lung collapse with stenosis of both the upper trachea and left main bronchus. She underwent successful bronchoscopic balloon and manual rigid tube dilatation with topical mitomycin-C application over the stenotic tracheal segment. A second bronchoscopic intervention was performed after 20 weeks for the left main bronchus stenosis with serial balloon dilatation and topical mitomycin-C application. These interventions led to significant clinical and radiographic improvements.

**Conclusion:**

This case highlights that balloon dilatation and topical mitomycin-C application should be considered in selected patients with tracheobronchial stenosis following endobronchial tuberculosis, avoiding airway stenting and invasive surgical intervention.

## Background

Endobronchial tuberculosis (EBTB) is a consequence of pulmonary TB (PTB) extending into the endobronchial or endotracheal wall, and its incidence has been reported to range from 6 to 50 % of cases [1–3]. EBTB can affect any region of the tracheobronchial tree, with higher rates of central EBTB (proximal to lobar bronchi) compared to segmental bronchi [3]. The true incidence and complications of EBTB are unknown as routine bronchoscopy is not performed in all patients with PTB.

The clinical presentation of EBTB can be non-specific with cough, dyspnoea and stridor; however patients may also be asymptomatic. Generally, stridor occurs when the proximal airway diameter in an adult diminishes to approximately 6 mm [4]. Bronchial stenosis and strictures are sequelae of EBTB and may develop in up to 95 % of cases despite adequate antituberculous therapy [5, 6]. Eradication of the tubercle bacilli is the primary goal of treatment for EBTB, similar to standard first line treatment approaches Corticosteroids have improved clinical outcomes when used as an adjunct therapy in children, however this has not been shown to prevent bronchostenosis in adults and clinical concerns about its immunosuppressive effects in this setting persist [7].

Despite antituberculous and steroid therapy, the development of bronchial stenosis or strictures is usually irreversible and therefore requires airway patency to be restored by either bronchoscopic or surgical interventions [8]. There are various bronchoscopic techniques including laser, balloon dilatation and stent insertion [9–11]. We report a female patient who had two previous episodes of tuberculosis complicated by multi-level fibrostenotic EBTB involving the left main bronchus and the trachea. The left lung was successfully expanded following serial endoscopic treatment with subsequent use of mitomycin-C preventing the need for an airway stent.

## Case presentation

A 24-year-old female presented with a 3-month history of worsening dyspnoea and stridor. She was unable to climb one flight of stairs or walk more than 50 meters on a flat surface, requiring domiciliary oxygen therapy. She also had significant loss of appetite and loss of weight. Her past medical history included acid-fast bacilli (AFB) smear-positive PTB in 2007 and laryngeal TB in 2013 for which she completed 6 months of standard anti-tuberculous (anti-TB) regimen on both occasions.

Respiratory examination revealed tracheal deviation to the left, decreased chest expansion, reduced vocal fremitus and dullness to percussion of the left lung. Oxygen saturation on room air was 95 %.

**Fig. 1:**
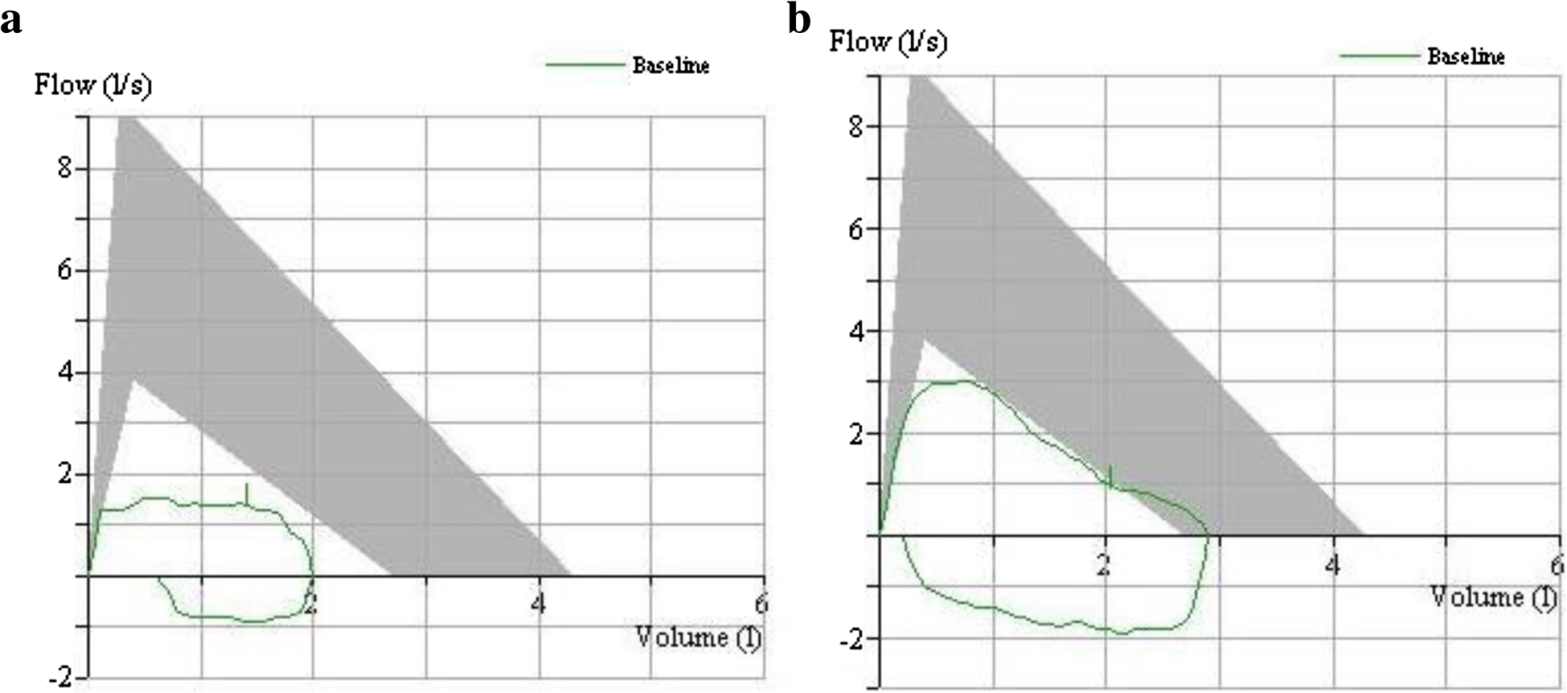
FV loop pre- and post-intervention. **a** FV loop showing flattening of inspiratory and expiratory limbs indicating fixed upper airway obstruction. **b** FV loop at 36 weeks following intervention showed improvement of both the inspiratory and expiratory limbs

**Fig. 2:**
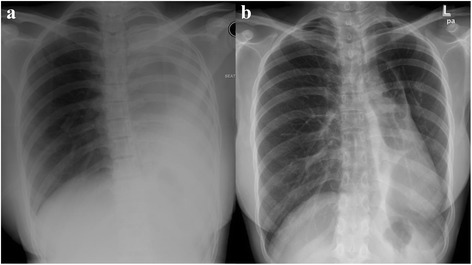
Chest radiograph pre- and post-intervention. **a** Chest radiograph showed total left lung collapse. **b** Chest radiograph at 36 weeks following intervention illustrating re-expansion of the left lung field

**Fig. 3:**
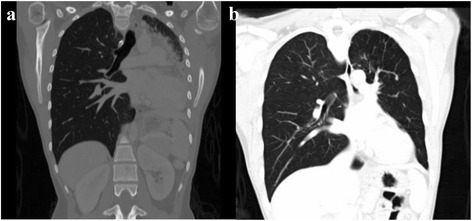
CT Thorax pre- and post-intervention. **a** Coronal view CT Thorax showed total left lung collapse. **b** Coronal view CT Thorax at 36 weeks following intervention showing re-expansion of the left lung

**Fig. 4:**
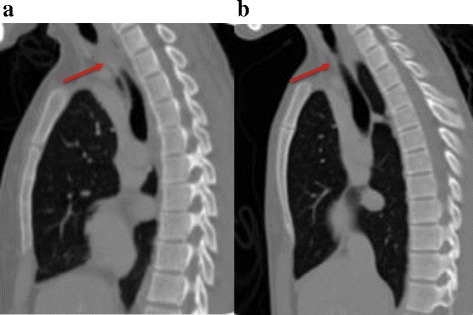
CT Thorax pre- and post-intervention. **a** Lateral view of a CT Thorax showing 1 cm of total stenosis at the upper one third of trachea. **b** Lateral view CT Thorax at 36 weeks following successful intervention

**Fig. 5:**
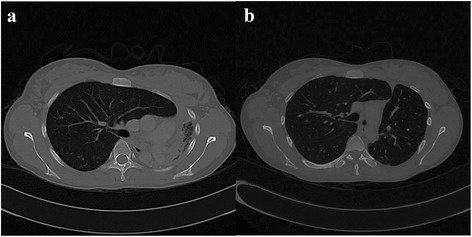
Axial view of a CT Thorax at the main carina level pre- and post-intervention. **a** CT Thorax showed left main bronchus stenosis with collapsed left lung. **b** CT Thorax at 36 weeks following intervention with residual left main bronchus stenosis and re-expansion of the left lung

**Fig. 6:**
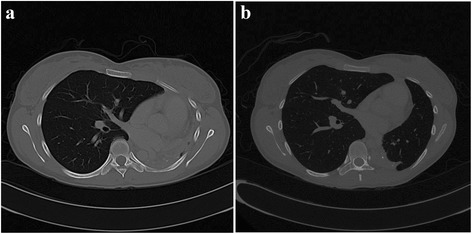
Axial view CT Thorax pre- and post-intervention. **a** CT Thorax showed mediastinal shift to the left. **b** CT Thorax at 36 weeks following intervention showed normal location of the mediastinum

On this presentation, sputum smear examination for AFB was negative. Flow volume (FV) loop was consistent with fixed airway obstruction (Fig. 1a) with her best functional vital capacity (FVC) of 2.03 litres (58 % predicted). Chest radiograph (Fig. 2a) and computed tomography (CT) scan of thorax (Fig. 3a) showed total left lung collapse. The CT thorax also revealed 1-cm segment of tracheal stenosis (Fig. 4a) and left main bronchus (LMB) stenosis (Fig. 5a) with mediastinal shift to the left (Fig. 6a).

Flexible bronchoscopy revealed a web-like 90 % tracheal stenosis and the visualisation of airways distally from this point was not possible. A clinical judgement of PTB reactivation was made based on the bronchoscopic appearance and the presence of constitutional symptoms. The standard four anti-TB regimen was therefore commenced. Patient was then referred to an interventional pulmonology centre for bronchoscopic intervention.

**Fig. 7:**
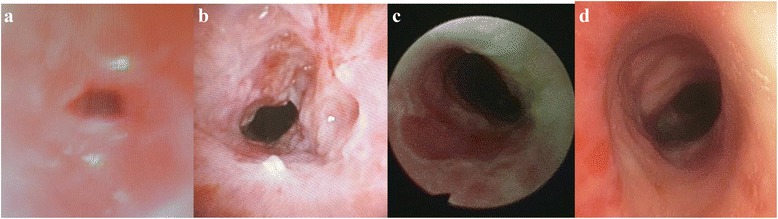
Bronchoscopic findings (**a**) during presentation showing stenotic upper tracheal segment. **b** 3 weeks **c** 8 weeks and **d** 16 weeks post-intervention

The first intervention performed was sequential tracheal dilatation to 10 mm via controlled radial expansion CRE® balloon (Boston Scientifics, USA) and mechanical dilatation with rigid bronchoscopy followed by mitomycin-C application (0.4 mg/ml) with a total contact time of 8 min. The first intervention was successful, as bronchoscopic surveillance performed at 3, 8 and 16 weeks (Fig. 7a) showed no recurrence of tracheal stenosis with a tracheal diameter of 10 mm. The airway distal to the tracheal stenosis became accessible which subsequently revealed the LMB stenosis with a pinpoint opening as previously reported in the CT thorax. We then proceeded with dilatation of the LMB.

The second intervention was performed at 20 weeks and the stenosed LMB was dilated using a CRE® balloon at 8, 9 and 10 mm. At 26 weeks, the balloon dilatation was repeated with the adjuvant treatment of topical mitomycin-C application (0.4 mg/mls) with the same contact time of 8 min. These procedures were repeated again at 36 weeks to re-examine further the LMB stenosis. There were no immediate complications.

Following these interventions, the patient showed significant and sustained improvement. The FV loop (Fig. 1b) illustrated improvement in both inspiratory and expiratory limbs and the FVC increased to 2.91 litres (83 % predicted). Chest radiograph (Fig. 2b) and CT thorax showed re-expansion of the left lung (Fig. 3b), tracheal patency (Fig. 4b) and the mediastinum back to its original position (Fig. 6b).

The patient is currently able to climb more than four flights of stairs and walk more than 1 kilometre without dyspnoea. She had completed a total 9 months of anti-TB therapy. She no longer requires oxygen and is back to her usual daily activities.

## Discussion

We report a rare case of multiple level tracheobronchial stenoses secondary to endobronchial tuberculosis. The risk factors present include female gender, symptom duration of more than 4 weeks and multiple previous episodes of PTB; these are significant predictors of EBTB [4]. Our patient had a unique presentation in which bronchoscopy revealed tracheal fibrostenosis as well as LMB involvement. These are both rare sites for EBTB as current literature reports that the right upper lobe and right main bronchus are the most common sites [5, 6, 12].

Although presentation of EBTB varies, patients are more commonly reported to present with cough and wheezing [13]. Localised rhonchi on respiratory examination should prompt the clinician to assess for the presence of an endobronchial lesion in which correlation of loop spirometry, CT findings and bronchoscopy may aid the diagnosis. The involvement of the trachea was the reason for the patient’s rare presentation of dyspnea and stridor.

Simple tracheal stenosis can be treated endoscopically while complex stenosis should be evaluated for surgery [8]. Various bronchoscopic techniques include laser, cryosurgery, controlled heat application, balloon dilatation and stent insertion are available [9–11]. Persistent airway stenosis following balloon dilatation has been described especially if active inflammation, calcification and malacia are evident. Patients who require more than one session of balloon dilatation may need more definitive treatment such as stenting or ablative procedures [10].

As there was no bronchomalacia evident in our patient, an airway stent was not inserted into the LMB to avoid long-term complications associated with this foreign device. This option would be considered however, if the LMB stenosis re-occurred following topical mitomycin-C application.

The use of mitomycin-C as an adjunct therapy to bronchoscopic procedures in our case was influenced by reports of successful treatment in patients with tracheal stenosis secondary to intubation [14, 15]. There are no controlled studies and very few case reports in the literature describing the utility of mitomycin-C for trachea-bronchial stenosis secondary to EBTB. Two previous reports had shown the success of topical mitomycin-C for obstructive granuloma and a 90 % occlusion of the upper trachea secondary to EBTB [16, 17]. Mitomycin-C is an antineoplastic antibiotic agent that inhibits fibroblast proliferation and modulates wound healing and scarring. Due to its teratogenic effects, use in a women of childbearing age woman should be coupled with contraceptive use.

This patient was successfully managed with clinical (symptoms and FV loop) and radiological improvements at short-term follow-up, despite having residual LMB stenosis. As there was no bronchomalacia, an airway stent was not inserted to the LMB.

## Conclusion

This case report contributes to the current literature and evidence base that balloon dilatation and topical mitomycin-C application should be considered in selected patients with tracheobronchial stenosis post EBTB. Importantly, this technique might obviate the need for airway stenting and further invasive surgical intervention.

### Consent

Written informed consent was obtained from the patient for publication of this case report.

### Availability of data and materials

All materials are included in this manuscript.

## Abbreviations

EBTB: endobronchial tuberculosis
PTB: pulmonary tuberculosis
LMB: left main bronchus
AFB: acid fast bacilli
FV: flow volume
FVC: functional vital capacity
CT: computed tomography
